# Development of a CT-Based comprehensive model combining clinical, radiomics with deep learning for differentiating pulmonary metastases from noncalcified pulmonary hamartomas: a retrospective cohort study

**DOI:** 10.1097/JS9.0000000000001593

**Published:** 2024-05-17

**Authors:** Yunze Liu, Hong Ren, Yanbin Pei, Leilei Shen, Juntang Guo, Jian Zhou, Chengrun Li, Yang Liu

**Affiliations:** a Medical School of Chinese General Hospital of PLA; b Department of Thoracic Surgery, First Medical Center, Chinese General Hospital of PLA, Beijing; c Department of Thoracic Surgery, Hainan Hospital of Chinese General Hospital of PLA, Sanya; d Department of Imaging, Changhai Hospital, Shanghai, People’s Republic of China

**Keywords:** CRDL, differentiation, noncalcified pulmonary hamartomas, pulmonary metastases

## Abstract

**Background:**

Clinical differentiation between pulmonary metastases and noncalcified pulmonary hamartomas (NCPH) often presents challenges, leading to potential misdiagnosis. However, the efficacy of a comprehensive model that integrates clinical features, radiomics, and deep learning (CRDL) for differential diagnosis of these two diseases remains uncertain.

**Objective:**

This study evaluated the diagnostic efficacy of a CRDL model in differentiating pulmonary metastases from NCPH.

**Methods:**

The authors retrospectively analyzed the clinical and imaging data of 256 patients from the First Medical Centre of the General Hospital of the People’s Liberation Army (PLA) and 85 patients from Shanghai Changhai Hospital, who were pathologically confirmed pulmonary hamartomas or pulmonary metastases after thoracic surgery. Employing Python 3.7 software suites, the authors extracted radiomic features and deep learning (DL) attributes from patient datasets. The cohort was divided into training set, internal validation set, and external validation set. The diagnostic performance of the constructed models was evaluated using receiver operating characteristic (ROC) curve analysis to determine their effectiveness in differentiating between pulmonary metastases and NCPH.

**Results:**

Clinical features such as white blood cell count (WBC), platelet count (PLT), history of cancer, carcinoembryonic antigen (CEA) level, tumor marker status, lesion margin characteristics (smooth or blurred), and maximum diameter were found to have diagnostic value in differentiating between the two diseases. In the domains of radiomics and DL. Of the 1130 radiomics features and 512 DL features, 24 and 7, respectively, were selected for model development. The area under the ROC curve (AUC) values for the four groups were 0.980, 0.979, 0.999, and 0.985 in the training set, 0.947, 0.816, 0.934, and 0.952 in the internal validation set, and 0.890, 0.904, 0.923, and 0.938 in the external validation set. This demonstrated that the CRDL model showed the greatest efficacy.

**Conclusions:**

The comprehensive model incorporating clinical features, radiomics, and DL shows promise for aiding in the differentiation between pulmonary metastases and hamartomas.

## Introduction

Highlights

- This is a multicenter study that used data from patients from more than one hospital, and achieved ideal results. It can reduces bias in our research, makes the conclusions more credible, and confirms the effectiveness of our model, which is beneficial for the promotion of the model and developing methods.
- Through clinical practice, we have observed that with the widespread use of computed tomography scans, many metastatic lung tumors are detected early. However, it can sometimes be challenging to distinguish these from noncalcifying teratomas based on the patient’s clinical features and radiological characteristics, potentially leading to detrimental impacts on the patient.
- In our study, we utilized a combination of clinical features (basic clinical data, laboratory indicators, and radiological semantic features), radiomics, and deep learning to differentiate between metastatic lung tumors and noncalcifying teratomas. Eventually we established a combined diagnostic model which named the CRDL model.
- The model has shown excellent discriminative performance. This provides substantial assistance to clinicians when they encounter difficulties in distinguishing between these two diseases.
- The datasets used and/or analyzed during the current study are available from the corresponding author on reasonable request.

Cancer is a leading cause of mortality worldwide, and remains a perennial subject of concern in medical research and patient care. The most recent Cancer Atlas indicates that in 2019, there were ~23.6 million (95% UI: 22.2–24.9 million) new cases of cancer and 10.0 million (95% UI: 9.36–10.6 million) cancer-related deaths globally^1^. According to ‘Cancer Statistics, 2024’ from CA, the estimated number of new cancer cases in the United States is 2 001 140 in 2024, with Lung and Bronchus cancer estimated to have 234 580 new cases^2^. One hallmark feature of cancer is its propensity for metastasis, which can dramatically decrease patient survival. Besides, in ‘Cancer Statistics, 2024’, it is estimated that ~1680 people die from cancer every day, of which ~340 die from lung cancer every day, making lung cancer still the deadliest form of cancer^2^. The lungs, which receive blood from both the pulmonary and bronchial arteries and are characterized by low-pressure blood flow, are frequent sites of metastatic deposits from malignant tumors^3^. Pulmonary metastases are commonly identified during postoperative follow-up for other tumors, and while some patients may exhibit respiratory symptoms^3^, the origins of these metastases often trace back to primary lesions in the colon, rectum, kidney, breast, prostate, and oropharynx^4,5^.

Computed tomography (CT) is instrumental in identifying pulmonary metastases^6^, with the most typical CT findings being single or multiple round or oval nodules within the lungs. These nodules tend to have smooth margins and regular shapes, with homogenous density. They are predominantly located in the middle and lower lung fields and near pleural surfaces^7^. Evidence suggests that patients with pulmonary metastases, who are suitable candidates for surgery, can experience significant survival benefits with aggressive surgical interventions^7^.

In contrast, pulmonary hamartomas (PH) are benign growths that are often asymptomatic^8^. The increased prevalence of chest CT has led to incidental findings of PH during routine health screening. Classic PH features ‘popcorn-like’ nodules with characteristic fat and calcification^9^. However, numerous reports have described atypical PH that do not exhibit calcification^9–13^, known as noncalcified pulmonary hamartomas (NCPH). Accurate detection of NCPH is crucial, as it can prevent unnecessary surgical interventions, thus offering significant clinical advantages.

Recently, the concept of ‘omics’ has increasingly permeated the realm of medicine, giving rise to the emerging field of ‘medomics’, with radiomics as a key component. Radiomics (RAD) involves the extraction and analysis of quantitative features from medical images using advanced mathematical algorithms and machine learning techniques, with the goal of addressing various medical challenges^14^. With the gradual study of radiomics, it is not only used for the overall presentation of lesions, but also for the diagnosis, staging, prognosis, and efficacy evaluation of diseases. Rossi *et al*.^15^ used radiomics model to predict the efficacy of immunotherapy for renal cell carcinoma and found that radiomics combined with other potential predictive factors can provide personalized treatment for patients with advanced renal cell carcinoma. In the differential diagnosis of pulmonary hamartoma, Habert *et al*. and Xiaohuang *et al*. both applied the radiomics to differentiate pulmonary hamartoma from carcinoid tumors, and had conclusion that radiomics signature had significance to distinguish two diseases^16,17^. The advantage of radiomics lies in its ability to reveal clinical outcomes through noninvasive means and guide clinical decision-making, providing new ways for disease diagnosis and treatment.

The development of robust radiomics-based conclusions typically requires extensive high-quality datasets, for which deep learning (DL) can provide substantial reinforcement. Unlike other analytical methods, the neural network architecture of DL allows models to expand exponentially in response to increasing data volumes and complexities, it is one part of machine learning^18^. DL is now commonly used in the medical field in combination with omics to analyze diseases, which is directly related to its applicability in big data processing. Wang *et al*.^19^ developed a DL-pathomics, radiomics, and immunoscore model to predict the postoperative OS and DFS of colorectal cancer lung metastasis patients, and showed reliable results. In order to integrate with clinical practice, many models established included DL were CRDL models, which is a comprehensive model combining clinical features, radiomics, and DL^20,21^. However, research specifically targeting the distinction between pulmonary metastases and NCPH remains limited. Our study delves into the clinical features and radiomics of pulmonary metastases and NCPH through the lens of big data and aims to develop a CRDL model that can effectively differentiate between these two conditions.

## Materials and Methods

### Patient population

This retrospective study respectively analyzed data from 256 patients in the First Medical Centre of the General Hospital of the People’s Liberation Army and 85 patients in Shanghai Changhai Hospital between 1st January 2018, and 1st August 2023. It has been approved by the Ethics Committee of the the First Medical Centre of the General Hospital of the People’s Liberation Army and Shanghai Changhai Hospital, and the approval number are S2023-388-01 with the First Medical Centre of the General Hospital of the People’s Liberation Army and CHEC2021-032 with Shanghai Changhai Hospital, respectively. The cohort of the First Medical Centre of the General Hospital of the People’s Liberation Army comprised 184 cases of hamartomas and 72 cases of metastatic tumors, meanwhile the cohort of Shanghai Changhai Hospital comprised 37 cases of hamartomas and 48 cases of metastatic tumors, all of which were pathologically confirmed after pulmonary surgery. Eligibility for inclusion in the study was based on the following criteria: 1) the patient had undergone a comprehensive chest CT scan within 2 weeks prior to surgery, 2) the quality of the imaging was satisfactory for analysis (i.e. free from motion artifacts), and 3) a definitive pathological diagnosis was available. Patients were excluded from the study if they 1) had undergone invasive procedures, such as puncture or radiofrequency ablation of the tubercle prior to surgery, which could potentially affect imaging; 2) lacked essential data, such as CT images or clinical records; and 3) presented with a pulmonary lesion that was not nodular in nature. This research protocol was approved by the ethics committees of the First Medical Centre of the General Hospital of the People’s Liberation Army and Shanghai Changhai Hospital. The work has been reported in line with the strengthening the reporting of cohort, cross-sectional, and case–control studies in surgery (STROCSS) criteria^22^ (Supplemental Digital Content 1, http://links.lww.com/JS9/C575).

### Clinical features assessment

The clinical features examined in this study included sex, age, BMI, initial time of discovery, presence or absence of respiratory symptoms, weight loss, smoking history, and family history of cancer as well as laboratory test results and conventional semantic features of CT scans. Laboratory tests included routine blood work and common lung cancer tumor markers, such as carcinoembryonic antigen (CEA), cytokeratin 19 fragment (CYFRA21-1), neuron-specific enolase (NSE), and Squamous Cell Carcinoma Antigen (SCC).

The conventional semantic features of CT scans were assessed by two experienced physicians, each with over 5 years of experience in imaging. These features included the lesion site (left upper, left lower, right upper, right middle, and right lower lobe), margin characteristics (smooth or blurred), maximum diameter, proximity to the pleura (yes/no), anatomical location (peripheral/central), nature of the nodule (solid or other), and number of nodules (single/multiple).

Additional clinical features were retrospectively obtained from the electronic medical records of the the First Medical Centre of the General Hospital of the People’s Liberation Army (PLA) and Shanghai Changhai Hospital. All clinical data are presented in Table S1 in the Supplemental Material (Supplemental Digital Content 2, http://links.lww.com/JS9/C576).

### Pathological diagnosis

The pathological diagnoses of both conditions are relatively straightforward and present little difficulty in differentiation. However, to ensure the reliability of the pathological results, the diagnoses of all the patients were retrospectively reviewed by pathologists with over a decade of experience at two hospitals. All the pathological results were confirmed to be reliable.

### CT image acquisition

Imaging was performed using a Philips Brilliance ICT 512-layer spiral CT scanner. The scan parameters were as follows: tube voltage of 120 kV, rotation speed of 0.2 s per rotation, collimation of 80 mm, scanning matrix of 512×512, standard slice thickness of 5 mm, reconstruction of 1.5 mm thin slices for both lung and mediastinal windows, and pitch of 0.75. The patients were positioned supine, with their arms resting above their heads, and inserted into the scanner.

### Procedures

**Figure 1:**
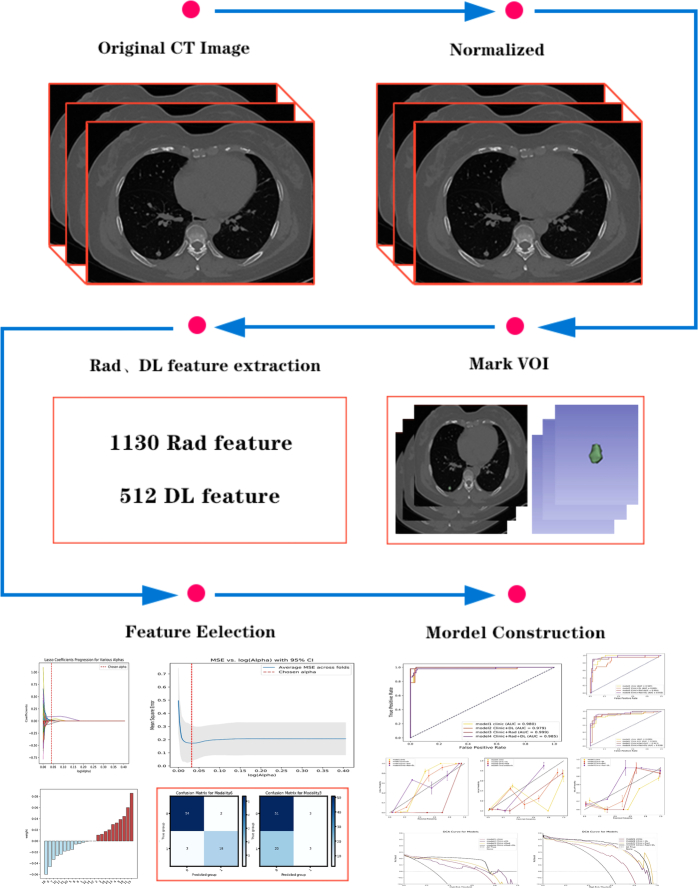
The clinical features, radiomics, and deep learning modeling pipeline.

The CRDL modeling pipeline is shown in Figure 1.

### Image preprocessing and segmentation

Following the acquisition of raw DICOM data from patients, format conversion, bias field correction, and normalization were performed on all CT images using an open-source Python 3.7 data package. These preprocessed images were then imported into 3D Slicer 5.2.2 software to adjust to the unified grayscale parameters.

Subsequent to this preprocessing, manual segmentation of 1.5 mm thin-layer images from preoperative plain CT scans was performed. Two physicians, both qualified in CT scan interpretation, meticulously delineated and preserved the region of interest (ROI) in each layer along the edge of the tubercle. This was achieved using the freehand sketching tool in the software, with images magnified to three times their original size and then combined to generate a three-dimensional volume of interest (VOI).

The two physicians were provided with 256 randomized datasets that were evenly split between them. Moreover, to mitigate bias, a minimum interval of 14 days was maintained between the segmentations of the two cases.

### Radiomic feature and DL extraction

Patients from the First Medical Centre of the General Hospital of the People’s Liberation Army (PLA) were randomly allocated to a training set and an internal validation set at a 7:3 ratio, and patients from Shanghai Changhai Hospital served as an external validation set. Subsequent to the spatial alignment of the sketched VOI with the raw DICOM files, radiomic features for three sets were extracted using an open-source Python 3.7 package. These features were categorized into shape, intensity, and texture groups, resulting in the extraction of 1130 features. Texture features were designed to characterize the relationships between pixels within the delineated regions and those in adjacent areas. To compute these features, we utilized the gray level co-occurrence matrix (GLCM), gray level size zone matrix (GLSZM), gray level run-length matrix (GLRLM), neighboring gray tone difference matrix (NGTDM), and gray level dependence matrix (GLDM).

Additionally, deep convolutional neural networks (DCNNs) have been developed using 3D-ResNet for advanced feature learning. The images and DICOMs were read and converted into standardized NumPy arrays, from which 512 features were extracted.

### Feature stability evaluation and radiomic signature building

Upon extraction of the radiomic features from the training set, the data were standardized. To harmonize the data delineations made by the two physicians, we employed the Z-score normalization method for three datasets. The intraclass correlation coefficient (ICC) was used to evaluate the consistency of the features derived from the physicians’ manual delineations. We retained only those features with an ICC greater than 0.75.

Subsequently, a correlation analysis was conducted and a correlation coefficient matrix was generated. Features demonstrating an absolute correlation coefficient exceeding 0.9 were excluded to address multicollinearity and to preliminarily select features with statistically significant differences using *t*-tests, rank sum tests, and correlation analyses.

The least absolute shrinkage and selection operator (LASSO) regression, along with fivefold cross-validation, was utilized to pinpoint highly predictive features. The dimensionality of the DL features was reduced using a similar approach. Consequently, we identified the premodelling features for radiomics and DL by setting the prefusion stage.

### Model development

Four models were developed in this study: 1) clinical features model (CM), 2) Clinic + DL-based model, 3) Clinic + Rad-based model, and 4) Clinic + Rad + DL model (CRDL). Five algorithms, namely, support vector machine (SVM), random forest (RF), K-nearest neighbor (KNN), stochastic gradient descent (SGD), and extreme gradient boosting (XGBoost), were applied to establish models separately. These algorithms have been widely applied in radiomics and have achieved a good performance in previous studies^23–26^.

We iteratively performed modeling and validation using the internal and external validation sets to ascertain the classifier with the highest diagnostic efficacy. Upon identifying this optimal classifier, we finalized and outputted the corresponding model.

### Statistical analyses

Statistical analyses were conducted using SPSS version 23.0 and Python 3.7 for feature extraction and statistical evaluation. Specifically, feature extraction, selection, model development, and testing were executed in Python, and comparisons of variable sets were performed using the SPSS software. Normally distributed measurement data are expressed as mean±SD (x̄±s), whereas non-normally distributed data are represented as median and interquartile range [M (Q1, Q3)]. Categorical data were presented as counts or percentages.

Comparative analyses among the training and validation sets employed the Student’s *t*-test for quantitative variables with semantic meaning and the *χ* ^2^ or Fisher’s exact test for qualitative variables. All statistical tests were two-sided, with a significance threshold of *P*<0.05.

The performance of the model was assessed using the receiver operating characteristic (ROC) curve, which included the sensitivity, specificity, accuracy (ACC), negative predictive value (NPV), positive predictive value (PPV), and area under the ROC curve (AUC) as evaluation metrics. Calibration curves were used to gage the concordance between the observed outcomes and the predictions of the CRDL model. Decision curve analysis (DCA) was used to ascertain the clinical utility of the model.

## Results

### Baseline characteristics

The training set included 128 cases of hamartoma and 51 cases of metastasis from the First Medical Centre of the General Hospital of the People’s Liberation Army (PLA). And the internal validation set comprised of 56 cases of hamartoma and 21 cases of metastasis, relatively, the external validation set comprised of 37 cases of hamartoma and 48 cases of metastasis. *χ* ^2^, Fisher’s exact, and Student’s *t*-tests revealed no significant differences in the clinical features between the training and internal validation set, except for age and family history of cancer. As for the training set and external validation set, there were significant differences in the laboratory indicators of blood routine except for platelets, which may be related to the different reagents and indicator units selected by different hospitals. Besides, there was no significant difference in other clinical features between the training set and the external validation set. Additionally, there was no statistical difference in the sample sizes between the training and internal validation set, indicating a balanced distribution of subgroup sample sizes. The clinical characteristics of the training and validation sets are presented in Table 1 and Table 2.

**Table 1:** Clinical features in the training and internal validation set.

Variable	Training set (*N*=179)	Internal validation set (*N*=77)	*P*
Anthropometric			
Age (years)	56.0 (49.0–61.0)	53.0 (46.0–58.0)	0.038
Sex			0.379
Female	103 (57.5)	39 (50.6)	
Male	76 (42.5)	38 (49.4)	
BMI	24.4 (22.5–26.4)	25.10 (22.50–26.67)	0.560
Earliest discovered time (months)	3.0 (1.0–12.0)	3.0 (1.0–12.0)	0.918
Symptoms of respiratory			0.587
Yes	17 (9.5)	5 (6.5)	
No	162 (90.5)	72 (93.5)	
Weight loss			0.678
Yes	6 (3.6)	1 (1.3)	
No	173 (96.6)	76 (98.7)	
Smoking history			0.988
Yes	47 (26.3)	21 (27.3)	
No	132 (73.7)	56 (72.7)	
History of cancer			0.700
Yes	50 (27.9)	19 (24.7)	
No	129 (72.1)	58 (75.3)	
Tumor therapy			1.000
Yes	30 (16.8)	13 (16.9)	
No	149 (83.2)	64 (83.1)	
Family history of cancer			0.046
Yes	42 (23.5)	9 (11.7)	
No	137 (76.5)	68 (88.3)	
RBC	4.43 (4.10–4.89)	4.60 (4.34–4.94)	0.063
WBC	5.82 (4.86–7.05)	5.77 (4.73–6.87)	0.822
N	0.586 (0.520–0.654)	0.566 (0.500–0.623)	0.194
L	0.314 (0.257–0.370)	0.341 (0.278–0.390)	0.396
M	0.066 (0.056–0.077)	0.064 (0.056–0.079)	0.353
PLT	215±58.2	215±43.8	0.911
Tumor marker			0.924
High	30 (16.8)	14 (18.2)	
Normal	149 (83.2)	63 (81.8)	
Semantic features			
Site			0.854
LU	41 (22.9)	19 (24.7)	
LL	38 (21.2)	15 (19.5)	
RU	48 (26.8)	16 (20.8)	
RM	5 (2.8)	4 (5.2)	
RL	47 (26.2)	23 (29.9)	
Margin			0.871
Smooth	115 (64.2)	51 (66.2)	
Blur	64 (35.8)	26 (33.8)	
Maximum diameter	13.0 (10.0–16.0)	12.0 (10.0–17.0)	0.782
Close to the pleura			0.495
Yes	63 (35.2)	23 (29.9)	
No	116 (64.8)	54 (70.1)	
Anatomic location			0.539
Peripheral	171 (95.5)	72 (93.5)	
Central	8 (4.5)	5 (6.5)	
Nodular nature			0.261
Solid	170 (95)	70 (90.9)	
Else	9 (5)	7 (9.1)	
Numbers			0.360
Single	166 (92.7)	68 (88.3)	
Multiple	13 (7.3)	9 (11.7)	
Pathological diagnosis		0.962	
Hamartoma	128 (71.5)	56 (72.7)	
Metastatic	51 (28.5)	21 (27.3)	

*P*-value refer to the results of Student’s *t*-test, *χ* ^2^ test, or F-test exact test when comparing continuous or categorical variables.

L, lymphocyte; LL, left lower lobe; LU, left upper lobe; M, monocyte; N, neureophil granulocyte; RL, right lower lobe; RM, right middle lobe; RU, right upper lobe.

**Table 2:** Clinical features in the training and external validation set.

Variable	Training set (*N*=179)	External validation set (*N*=85)	*P*
Anthropometric			
Age (years)	56.0 (49.0–61.0)	58.0 (54.0–65.0)	0.001
Sex			0.039
Female	103 (57.5)	38 (44.7)	
Male	76 (42.5)	47 (55.3)	
BMI	24.4 (22.5–26.4)	23.92 (21.64–26.57)	0.268
Earliest discovered time (months)	3.0 (1.0–12.0)	6.0 (1.0–12.0)	0.518
Symptoms of respiratory			0.058
Yes	17 (9.5)	15 (17.6)	
No	162 (90.5)	70 (82.4)	
Weight loss			0.181
Yes	6 (3.6)	0 (0)	
No	173 (96.6)	85 (100)	
Smoking history			0.948
Yes	47 (26.3)	22 (25.9)	
No	132 (73.7)	63 (74.1)	
History of cancer			<0.001
Yes	50 (27.9)	47 (55.3)	
No	129 (72.1)	38 (44.7)	
Tumor therapy			
Yes	30 (16.8)		
No	149 (83.2)		
Family history of cancer			0.006
Yes	42 (23.5)	8 (9.4)	
No	137 (76.5)	77 (90.6)	
RBC	4.43 (4.10–4.89)	5.34 (4.32–6.35)	<0.001
WBC	5.82 (4.86–7.05)	4.42 (3.95–4.68)	<0.001
N	0.586 (0.520–0.654)	3.26 (2.40–3.93)	<0.001
L	0.314 (0.257–0.370)	1.44 (1.12–1.87)	<0.001
M	0.066 (0.056–0.077)	0.39 (0.31–0.49)	<0.001
PLT	215±58.2	195±60.3	0.008
Tumor marker			0.018
High	30 (16.8)	25 (29.4)	
Normal	149 (83.2)	60 (70.6)	
Semantic features			
Site			0.005
LU	41 (22.9)	14 (16.5)	
LL	38 (21.2)	16 (18.8)	
RU	48 (26.8)	19 (22.4)	
RM	5 (2.8)	13 (15.3)	
RL	47 (26.2)	23 (27.0)	
Margin			0.505
Smooth	115 (64.2)	51 (60.0)	
Blur	64 (35.8)	34 (40.0)	
Maximum diameter	13.0 (10.0–16.0)	14.0 (10.0–16.0)	0.370
Close to the pleura			0.096
Yes	63 (35.2)	39 (45.9)	
No	116 (64.8)	46 (54.1)	
Anatomic location			0.762
Peripheral	171 (95.5)	80 (94.1)	
Central	8 (4.5)	5 (5.9)	
Nodular nature			0.023
Solid	170 (95)	74 (87.1)	
Else	9 (5)	11 (12.9)	
Numbers			
Single	166 (92.7)		
Multiple	13 (7.3)		
Pathological diagnosis		<0.001	
Hamartoma	128 (71.5)	37 (43.5)	
Metastatic	51 (28.5)	48 (56.5)	

*P*-value refers to the result of Student *t*-test, *χ* ^2^, or F-test exact test when comparing continuous or categorical variables.

L, lymphocyte; LL, left lower lobe; LU, left upper lobe; M, monocyte; N, neureophil granulocyte; RL, right lower lobe; RM, right middle lobe; RU, right upper lobe.

### Radiomics and clinical signature

During the signature construction phase, we initially developed seven clinical signatures [encompassing white blood cell count (WBC), platelet count (PLT), history of cancer, carcinoembryonic antigen (CEA), tumor marker status, margin characteristics (smooth or blurred), and maximum tumor diameter)] for the clinical model (CM) following dimensionality reduction. Subsequently, we utilized 24 radiomic signatures to develop CT radiomics-based models and seven deep-learning signatures to construct deep-learning-based CT models. Finally, we created three postfusion models, Clinic + Radiomics, Clinic + DL, and Clinic + Radiomics + Deep Learning (CRDL), by fusing probabilities derived from their respective model sets using the RF algorithm. Additionally, for comparative purposes, we employed five different algorithms: RF, SVM, KNN, XGBoost, and SGD.

### Model performance

**Figure 2:**
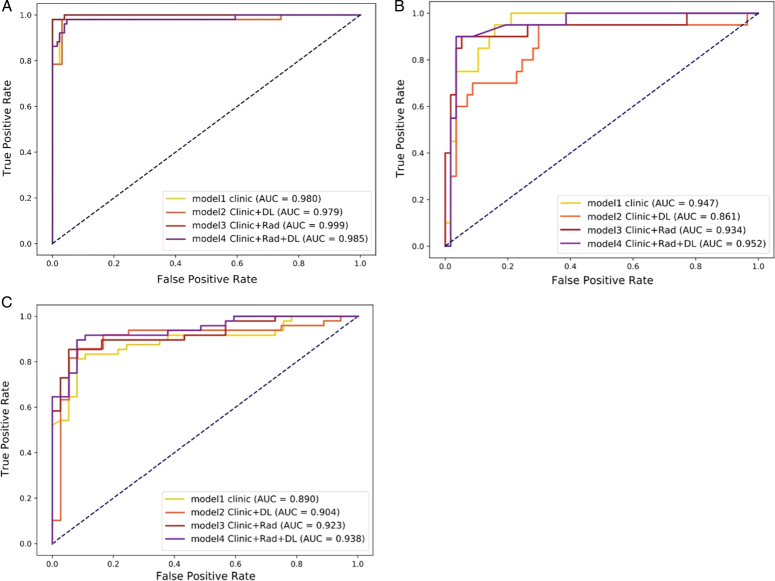
Receiver operation characteristic (ROC) curves of postfusion models in the training set (A), internal validation set (B) and external validation set (C). The clinical features, radiomics, and deep learning model had the best discriminating ability among four models, with an area under the curve (AUC) of 0.985 in the training set, 0.952 in the internal validation set and 0.938 in the external validation set.

Ultimately, the SVM model has emerged as the one with the most robust diagnostic capabilities. As shown in Table 3, all four models demonstrated commendable efficacy in distinguishing between pulmonary metastases and NCPH. Notably, the CRDL model outperformed the other models on three sets. The ROC curves for the four models on the training and two validation sets are shown in Figures 2A, B, and C, respectively. The area under the ROC curve (AUC) values for the four groups were 0.980, 0.979, 0.999, and 0.985 in the training set, and 0.947, 0.816, 0.934, and 0.952 in the internal validation set, and 0.890, 0.904, 0.923, and 0.938 in the external validation set. These results indicate that each model exhibits a good level of performance with particularly strong efficiency in the training set.

**Table 3:** Performance of four models in the training set, internal validation set, and external validation set.

Group	Model	AUC	Sensitivity	Specificity	ACC	NPV	PPV
Training set	CM	0.980	97.6%	88.9%	95.0%	0.941	0.953
	Clinic+DL	0.979	97.6%	88.9%	95.0%	0.941	0.953
	Clinic+Rad	0.999	96.0%	86.8%	93.3%	0.902	0.945
	CRDL	0.985	96.8%	94.7%	94.4%	0.922	0.953
	CM	0.947	96.0%	66.7%	85.7%	0.900	0.842
Internal validation set	Clinic+DL	0.861	88.7%	58.3%	79.2%	0.700	0.825
	Clinic+Rad	0.934	96.2%	75.0%	89.6%	0.900	0.895
	CRDL	0.952	96.5%	90.0%	94.8%	0.900	0.965
	CM	0.890	81.8%	69.8%	72.9%	0.916	0.486
External validation set	Clinic+DL	0.904	87.1%	83.3%	84.7%	0.918	0.750
	Clinic+Rad	0.923	85.3%	84.3%	84.7%	0.896	0.784
	CRDL	0.938	87.2%	93.5%	93.5%	0.896	0.919

ACC, accuracy; AUC, area under the receiver operating characteristic curve; CM, clinical model; CRDL, clinical and radiomics and deep learning; NPV, negative predictive value; PPV, positive predictive value.

The sensitivity of the CRDL model was 96.8% in the training set, 96.5% in the internal validation set, and 87.2% in the external validation set, whereas the specificities were 94.7%, 90.0% and 93.5% in three sets, respectively. The comprehensive statistical results of the other three models are presented in Table 3. Clinical models can be effective in discriminating between the two diseases, while the CRDL model further enhances diagnostic efficacy.

**Figure 3:**
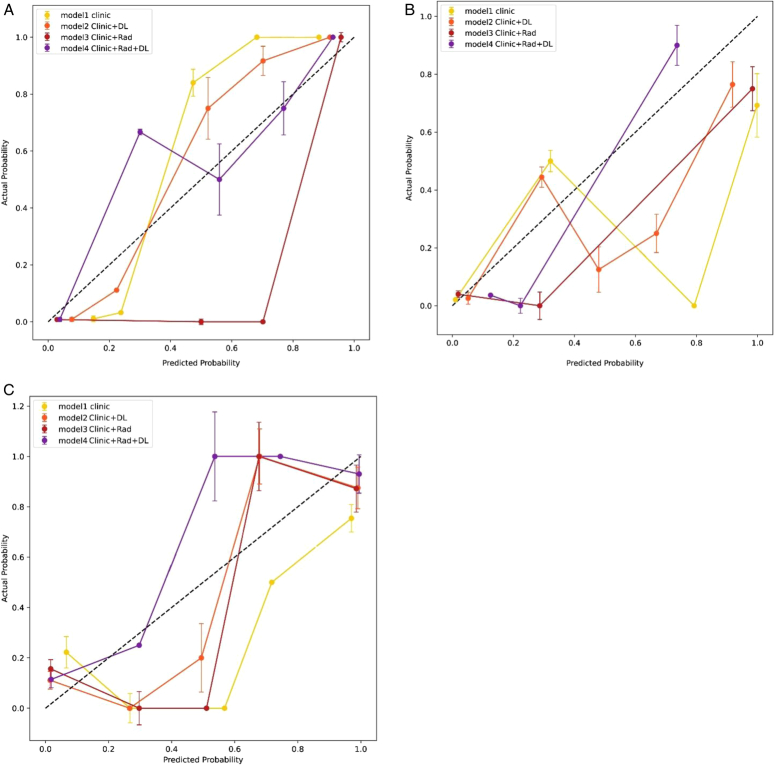
The calibration curves of postfusion models in the training set (A), internal validation set (B), and external validation set (C). The *x*-axis is the predicted probability calculated by the models, and the *y*-axis is the actual probability. The black dotted line means an ideal evaluation by the perfect model, the solid lines with different colors show the discrimination ability of different models. Which line is closer to the dotted line has better performance.

The actual pathological diagnoses agreed with the predictions made by the CRDL model for the training and validation sets. The calibration curves for the postfusion models in three sets are presented in Figure 3, with all sets exhibiting a clear trend.

**Figure 4:**
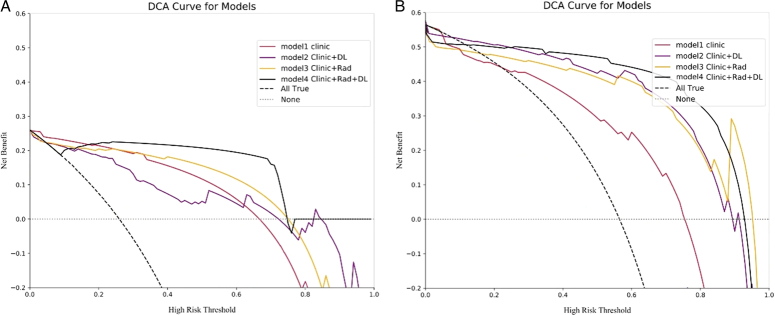
Decision curve analysis (DCA) of the postfusion models in the internal validation set (A) and external validation set (B). The *x*-axis means the high risk threshold, and the *y*-axis means clinic net benefit.

DCA was used to assess the discriminative properties of these models to evaluate their clinical applicability. The CRDL model demonstrated a net clinical benefit in distinguishing between pulmonary metastases and NCPH, indicating that it offers substantial clinical utility. The DCA for the postfusion models in the validation sets are shown in Figure 4A and B.

## Discussion

The clinical features have good diagnostic validity in distinguishing between lung metastases and NCPH, and the validity can be further enhanced when supplemented with Radiomics and DL results. Nevertheless, misdiagnosis of these two nodule types still occurs in clinical practice, and there is a marked difference in the treatment and prognosis between them. An accurate diagnosis would undoubtedly be of great benefit to patients.

Many patients can form preliminary conclusions about these diseases based on clinical and imaging factors because of the stark differences in their nature. Patients with metastatic lung cancer, similar to metastases in other regions, may benefit from specific diagnostic markers (such as tumor markers) for diagnosis. The imaging characteristics include smooth margins, multiplicity, and rapid growth. However, with the widespread adoption of CT, many early stage pulmonary metastases have been detected when serological and imaging indicators are not yet definitive. In some cases, the primary lesion was not identified before the discovery of pulmonary metastases. During the collection of medical records for this study, we identified more than eight cases of pulmonary metastases, mostly originating from gastrointestinal sources, where the primary tumor was not detected before surgery.

To further enhance the differential diagnostic capability for these two conditions, we employed Python 3.7 software to analyze the clinical and imaging data of the patients. The highest-performing single model was based on clinical features and aligned with our clinical experience. As a benign tumor, hamartoma presents a significant clinical distinction from malignant tumors, particularly in advanced and metastatic stages. The diagnostic focus is primarily on oncological markers and a history of cancer. Prior research by Higashi, Satoh, and Sánchez highlighted the remarkable diagnostic utility of tumor markers, such as CEA, CYFRA-21-1, and SCC, particularly in advanced and metastatic tumors. Elevated tumor marker levels, especially when several times higher than the upper limit of normal values, can be a significant indicator of tumor recurrence and metastasis^27–29^.

However, in previous research on lung metastases, no one has differentiated them from any particular benign disease. Studies have indicated that pulmonary metastases are frequently multiple, spherical, varying in size, and often located in the outer third of the lungs, typically without clinically significant symptoms. In contrast, this study specifically selected NCPH for their imaging resemblance to pulmonary metastases, thus making the research more focused and relevant. In clinical practice, it is challenging to differentiate between these two diseases.

In addition, this study improved the diagnostic process by combining radiomics and DL methods with traditional clinical indicators. We explored the integration of the three models and found that the CRDL model demonstrated superior efficacy in the differential diagnosis of two diseases over the other models. These findings suggest that Radiomics and DL can be used as important adjuncts to clinical models to significantly improve diagnostic accuracy.

### Advantages

This study distinguishes itself from previous radiomic research with four notable advantages. First, this is a multicenter study that used data from patients from more than one hospital, and achieved ideal results. It can reduces bias in our research, makes the conclusions more credible, and confirms the effectiveness of our model, which is beneficial for the promotion of the model and developing methods.

Second, the widespread use of chest CT in routine medical practice has led to an increase in the detection of early stage metastatic lung tumors, many of which lack identifiable primary lesions. These cases present a diagnostic challenge, as they can be difficult to differentiate from noncalcified hamartomas. Given the scarcity of research focused on the differential diagnosis of pulmonary metastases and NCPH, the innovative nature of this study is clear.

Third, in clinical practice, our assessment of pulmonary nodules is based on a combination of clinical and radiographic characteristics. We systematically classified these attributes and integrated radiomic signatures to extract more nuanced information from CT data. This methodology has uncovered features with potent discriminative capabilities between the two conditions, which lend itself to clinical interpretation. Consequently, the model demonstrated impressive diagnostic effectiveness.

Finally, the incorporation of DL tools into the model rendered the results more objective. This technological advancement enhances the performance of the model, providing a more sophisticated and unbiased analysis of the data.

### Limitations

This study has some limitations. On the one hand, the retrospective nature of this study could have introduced bias in the collection of clinical features. A prospective study would allow for more comprehensive model validation. On the other hand, given the constraints of real-world studies, the number of samples available for analysis was limited. To reinforce our findings, we contemplate extending the duration of the study.

## Conclusion

The proposed CT-based CRDL model demonstrates substantial potential for distinguishing between pulmonary metastases and NCPH. Its implementation may substantially impact clinical decision-making when encountering nodules of an indeterminate nature. With this model, patients with pulmonary metastases could receive timely treatment, while those with PH might avoid unnecessary surgery and opt for periodic monitoring. Therefore, the clinical utility of this model was evident.

## Ethical approval

The study was approved by the Ethics Committees of the General Hospital of the Chinese People's Liberation Army (approval number: S2023-388-01) and Changhai Hospital of Shanghai (approval number: CHEC2021-032).

## Consent

This study is a retrospective cohort study. Informed consent was waived by our ethics committee because of the retrospective nature of our study, and which will not have adverse effects on the health and rights of patients.

## Sources of funding

This study was supported by a grant from the Beijing Capital Clinical Characteristic Diagnosis and Treatment Technology Research Project (Z221100007422124).

## Author contribution

Y.L.: project administration and supervision; Y.Z.L.: conceptualization, data curation, formal analysis, funding acquisition, investigation, methodology, resources, software, validation, visualization, and writing – original draft; H.R.: writing – original draft and writing – review and editing; Y.B.P., L.L.S., and J.Z.: writing – review and editing; J.T.G.: supervision and writing – review and editing; C.R.L.: funding acquisition and writing – review and editing.

## Conflicts of interest disclosure

The authors declare that they have no financial conflict of interest with regard to the content of this report.

## Research registration unique identifying number (UIN)

1. Name of the registry: not applicable.
2. Unique identifying number or registration ID: not applicable.
3. Hyperlink to your specific registration (must be publicly accessible and will be checked): not applicable.

## Guarantor

Yang Liu and Yunze Liu.

## Data availability statement

The datasets used and/or analyzed during the current study are available from the corresponding author on reasonable request.

## Provenance and peer review

Not commissioned, externally peer-reviewed.

## Supplementary Material
